# Supplementary material for: Polyamines and Their Biosynthesis/Catabolism Genes Are Differentially Modulated in Response to Heat Versus Cold Stress in Tomato Leaves (Solanum lycopersicum L.)
Source: Cells. 2020 Jul 22;9(8):1749. doi: 10.3390/cells9081749 (PMC7465501; doi:10.3390/cells9081749)

Supplementary Information

Polyamines and their biosynthesis/catabolism genes are differentially modulated in response to heat versus cold stress in tomato leaves (*Solanum lycopersicum* L.)

Rakesh K. Upadhyay[1, 2], Tahira Fatima[2], Avtar K. Handa[2], Autar K. Mattoo[1]*

[1]Sustainable Agricultural Systems Laboratory, United States Department of Agriculture, Agricultural Research Service, Henry A. Wallace Beltsville Agricultural Research Center, Beltsville, MD 20705-2350, USA

[2]Center of Plant Biology, Department of Horticulture and Landscape Architecture, Purdue University, W. Lafayette, IN 47907, USA

*Correspondence: Autar K. Mattoo (autar.mattoo@usda.gov)

Supplementary Tables

Table S1: The sequence identities of PA metabolic pathway genes identified from tomato genome in this study.

Table S2: List of genes and their primer sequences used for quantitative real– time PCR (qRT-PCR) analysis.

Table S3: Pearson correlation analyses among the levels of different forms of PAs during the cold or heat stresses

Table S4: Correlation among transcript levels of PA metabolic genes with free, conjugated and bound putrescine, spermidine and spermine

Table S5: Correlation among transcript levels of selected genes with free, conjugated and bound putrescine, spermidine and spermine

Table S6: Correlation among transcript levels of PA metabolic genes

Table S7: Correlation among transcript levels of PA metabolic genes and selected genes as shown

Table S8: Correlation among transcript levels of genes selected for comparison

Table S9: Statistically significant differences (p-value) among heat response genes expression studies and polyamines accusation

Table S10: Statistical significance differences (p-value) in cold response time points among genes expression studies and polyamines accusation

Figure S1: qRT-PCR of genes which encode proteins involved in photosynthetic machinery like nuclear encoded light-harvesting chlorophyll a/b-binding genes *LHCB1-6* and plastid encoded PSII complex gene psbA and accD genes in response to heat stress.

Figure S2: qRT-PCR of genes which encode proteins involved in photosynthetic machinery like nuclear encoded light-harvesting chlorophyll a/b-binding genes *LHCB1-6* and plastid encoded PSII complex gene psbA and accD genes in response to cold stress.

Figure S3: qRT-PCR of gene expression of phosphoenolpyruvate carboxylase (*PEPC*) and Isocitrate dehydrogenase (*ICDH*) genes in response to heat or cold stress

Figure S4: qRT-PCR mediated gene expression of ornithine decarboxylase (*OAT1)* gene in response to heat or cold stress

Figure S5: PCA analyses of active and variable parameters during heat stress.

Figure S6: PCA analyses of active and variable parameters during cold stress.

Figure S7: Cytoscape analyses of Pooled data from heat stress

Figure S8: Cytoscape analyses of Pooled data from cold stress

Table S1: The sequence identities of PA metabolic pathway genes identified from tomato genome in this study.

Process	Polyamine Pathway Enzymes	Gene Name	SGN ID	mRNA size (nt)
Biosynthesis	Arginase (EC 3.5.3.1)	*SlARG1*	Solyc01g091160.2.1	1529
		SlARG2	Solyc01g091170.2.1	1405
	Arginine decarboxylase (EC 4.1.1.9)	*SlADC1*	Solyc10g054440.1.1	2124
		SlADC2	Solyc01g110440.2.1	3558
	Ornithine decarboxylase (EC 4.1.1.17)	*SlODC1*	Solyc04g082030.1.1	1296
		SlODC2	Solyc03g098300.1.1	1167
	N-carbamoylputrescine amidohydrolase (EC 3.5.1.53)	*SlCPA*	Solyc11g068540.1.1	903
	Agmatine iminohydrolase /Agmatine deiminase EC 3.5.3.12)	*SlAIH*	Solyc12g038970.1.1	1128
	S-adenosylmethionine decarboxylase (EC 4.1.4.50)	*SlSamDc1*	Solyc05g010420.1.1	1083
		SlSamDc2	Solyc02g089610.1.1	1089
		SlSamDc3	Solyc01g010050.2.1	3606
	Spermidine synthase (EC 2.5.1.16)	*SlSPDS1*	Solyc05g005710.2.1	1319
		SlSPDS2	Solyc04g026030.2.1	1240
	Spermine synthase (EC 2.5.1.22)	*SlSPMS*	Solyc08g061970.2.1	1267
Catabolism	Flavin dependent polyamine oxidase (EC 1.5.3.11)	*SlPAO4-Like*	Solyc02g081390.2.1	2082
		SlPAO2	Solyc01g087590.2.1	1850
	Copper dependent amine oxidase (EC 1.4.3.6)	*SlCuAO*	Solyc08g079430.2.1	2369
		SlCuAO-Like	Solyc05g013440.2.1	2733

Table S2: List of genes and their primer sequences used for quantitative real– time PCR (qRT-PCR) analysis in this study.

Gene Name	SGN ID	Forward Primer (5'-3')	Reverse Primer (5'-3')
SlARG1	Solyc01g091160	TACTGCCAAGATATCCAAGTGACC	CCTCCCCAGTAAAGATGAGTACAA
SlARG2	Solyc01g091170	GTAAGAGAACTTGCTGCCAAGATG	ATCTGCCAGACCTACCTAATGTGA
SlADC1	Solyc10g054440	CTCGGCGGACTCCATAACC	GCCCAGGGACTGCATAGGT
SlADC2	Solyc01g110440	GGAGAAGGCGAAGGTGTTG	ATTGGTATTGGTATTGGCATTGG
SlODC1	Solyc04g082030	TGCGAGCTTTTGCTTCGAAT	GGTAATGCGCCGTATTTTGG
SlODC2	Solyc03g098300	ACGAAAGAAAGTTGTGAAATACG	CAAGATAGGTAGGTGTGGATAAC
SlAIH	Solyc12g038970	GGTGCAATTATTACTCCGCAATTT	CAATTTCCCTAGCACCTTCAATTC
SlCPA	Solyc11g068540	CGAGGGCTATTACTTTTGTCAAGC	CTACTCCAAGCTCCTTTGCAAGAT
SlSAMDc1	Solyc05g010420	CCTGGTGCTCAATCGTTTCC	TACCGGCAGCAAGCTTTCC
SlSAMDc2	Solyc02g089610	GCAATGATCGAGAGGGTGTTG	GGCCAGACTCGGTGTAAAGTTC
SlSAMDc3	Solyc01g010050	CCCGTGGGAGCTTCATCTT	GCTATCAAGGACCGCAACTTCT
SlSPDS1	Solyc05g005710	GGAGGAGGAGATGGTGGTGTCC	GCAACTCCGTCACCAATGTGGAGAT
SlSPDS2	Solyc04g026030	AAGGCTCTGTCAACTATGC	GGTGTGGCTATCGTCAAC
SlSPMS	Solyc08g061970	GATACTGGCATCTTCGTAACTC	GCTTGGTTGTGTTGTAAATG
SlPAO2	Solyc01g087590	TGATGGAAGGAAGAATGAGAG	CCAGCAGAGGTTCACTTAG
SlPAO4-like	Solyc02g081390	CCACTTCATATGCTTGCGGTTA	TCGAGGTCACAAGCAAGTCTTC
SlCuAO4	Solyc08g079430	CGATTTCCCCAATCATCCTTT	CCGCAATTGAATGAACGATTT
SlCuAO4-like	Solyc05g013440	CAATCGCACTGGGCAGTTAA	CTCCTCAAGAATTTTGCCTCTGA
SlTIP41	Solyc10g049850	AACCACATTTCAGGCCTTGTCTT	CATGGAGTTTTTGAGTCTTCTGCAT
SlUBI3	Solyc01g056940	TCGTAAGGAGTGCCCTAATGCTGA	CAATCGCCTCCAGCCTTGTTGTAA
SlHSP17.6	Solyc06g076560	AGGTCAAGTCCATTGAGATTTCTG	ACAGAGCAACTTCAAACCTTCAAC
SlHSP20.0	Solyc06g076570	AAGATTTAGACTTCCGGAGAATGC	GCCCATACAAGGATACAATAAATTCACAT
SlHSP20.1	Solyc06g076540	CTGTTCCAAAGGAAGAAGTGAAGA	AGAAACAGCGACAACTCAAACACT
SlCBF1	Solyc03g026280	TCAGGCCGTCGAAATCTTCC	ATGTAGGCATCAGTTTCCACACA
SlCBF2	Solyc03g124110	ACTTCGTGGATGAGGAAGCG	GGCATATAAGCGTGCACATCAG
SlCBF3	Solyc03g026270	CGCCGAAATCTTCCGACCTT	ACCCGGCATGCAGAATAACG
SlLHCB1	Solyc03g005770	AGTGAAGGAGATCAAGAACGGCA	GTCAGCAATGTGGTCGGCAA
SlLHCB2	Solyc12g006140	GTGTCACCATGCGACGTACTG	GTCCAGCAGTGTCCCATCCA
SlLHCB3	Solyc07g063600	CTCTTGAGGTTATCCATGGGAGATG	AGTCCAGCCCACCTTCACTG
SlLHCB4	Solyc09g014520	GATCCATTGGGCCTTGCTGC	CAAGAACGCAACCATGGCGA
SlLHCB5	Solyc06g063370	TTGGTGCCAACTGTGGTCCT	AACCTCTGCGACGACAGCAA
SlLHCB6	Solyc01g105050	TGAGGCTGGACAAGGGAAGAC	ACACTCAACGGCTCTTTCACA
SlICDH	Solyc11g011930	GGAGAGTTCATCGATGCTGA	TCTGACACCTTAATCCCAAACA
SlPEPC	Solyc07g062530	ATGAAAGGTATTGCTGCTGGA	TCGAGAAGCTACTAAACAAAGAGG
SlpsbA	NC_007898.3	ATATCGTAGCCGCTCATGGTTATT	CAGATACCTACTACAGGCCAAGCA
SlaccD	NC_007898.3	AAGGTTCACAAGCAGCTGAATACT	CGTGGAGCTTAAATAACTCACTCAG

Table S3: Pearson correlation analyses among the levels of different forms of PAs during the cold or heat stresses

**Table S2**	PUT-F	SPD-F	SPM-F	PUT-C	SPD-C	SPM-C	PUT-B	SPD-B	SPM-B
**PUT-FC**	**1.0**	**-1.0**	**-1.0**		**-0.9**	**-1.0**	**0.9**	**1.0**	**-0.8**
**PUT-FH**	**1.0**		**-0.9**	**0.9**	**0.9**	**-0.8**	**-0.8**	**0.8**	
**SPD-FC**	**-1.0**	**1.0**	**1.0**		**0.9**	**1.0**	**-0.9**	**-1.0**	**0.8**
**SPD-FH**		**1.0**		**0.8**	**0.8**				
**SPM-FC**	**-1.0**	**1.0**	**1.0**		**0.9**	**1.0**	**-0.8**	**-0.9**	
**SPM-FH**	**-0.9**		**1.0**	**-1.0**	**-0.9**	**0.9**		**-0.9**	
**PUT-CC**				**1.0**					
**PUT-CH**	**0.9**		**-1.0**	**1.0**	**1.0**	**-1.0**		**1.0**	
**SPD-CC**	**-0.9**	**0.9**	**0.9**		**1.0**	**1.0**	**-1.0**	**-1.0**	**0.9**
**SPD-CH**	**0.9**	**0.8**	**-0.9**	**1.0**	**1.0**	**-0.8**		**0.9**	
**SPM-CC**	**-1.0**	**1.0**	**1.0**		**1.0**	**1.0**	**-0.9**	**-1.0**	**0.8**
**SPM-CH**	**-0.8**		**0.9**	**-1.0**	**-0.8**	**1.0**	**0.8**	**-1.0**	
**PUT-BC**	**0.9**	**-0.9**	**-0.8**		**-1.0**	**-0.9**	**1.0**	**1.0**	**-1.0**
**PUT-BH**	**-0.8**					**0.8**	**1.0**		
**SPD-BC**	**1.0**	**-1.0**	**-0.9**		**-1.0**	**-1.0**	**1.0**	**1.0**	**-0.9**
**SPD-BH**	**0.8**		**-0.9**	**1.0**	**0.9**	**-1.0**		**1.0**	
**SPM-BC**					**0.9**	**0.8**	**-1.0**	**-0.9**	**1.0**
**SPM-BH**									**1.0**

NOTE: Red and Blue number indicate positive ($\geq$ 0.8) and negative ($<$ 0.8) Pearson correlation coefficients, respectively. C and B at the end of PAs abbreviations shows cold or heat stress, respectively.

Table S4: Correlation among transcript levels of PA metabolic genes with free, conjugated and bound putrescine, spermidine and spermine

Table S3	PUT-F	SPD-F	SPM-F	PUT-C	SPD-C	SPM-C	PUT-B	SPD-B	SPM-B
ARG1-C									
ARG1-H	-0.9		1.0	-0.9	-0.8	0.9	0.8	-1.0	
ARG2-C									
ARG2-H			0.9	-0.8	-0.9				
ADC1-C	0.8	-0.8	-0.8		-0.9	-0.8	0.8	0.9	-0.8
ADC1-H									
ADC2-C					-0.8	-0.8	0.8	0.8	
ADC2-H									
ODC1-C	0.9	-0.9	-0.9		-1.0	-0.9	1.0	1.0	-0.9
ODC1-H		0.8							
ODC2-C	-1.0	1.0	1.0		0.8	0.9		-0.9	
ODC2-H	-0.9		0.8	-0.8			0.9		
AIH1-C									
AIH1-H			0.9	-0.8		0.8		-0.9	
CPA-C					-0.8		0.9		-0.9
CPA-H			0.9	-0.8				-0.8	
SAMDC1-C					-0.8	-0.8		0.8	
SAMDC1-H									
SAMDC2-C	0.8	-0.8	-0.8		-0.8	-0.8	0.8	0.8	-0.8
SAMDC2-H									
SAMDC3-C									
SAMDC3-H									-0.9
SPDS1-C					-0.8		0.8	0.8	-0.8
SPDS1-H			0.8						
SPDS2-C									
SPDS2-H		0.8							
SPMS-C									
SPMS-H	0.9		-1.0	1.0	1.0	-0.9		0.9	
PAO2-C	0.9	-0.9	-0.8		-0.9	-0.9	0.9	0.9	-0.9
PAO2-H									
PAO4l-C									
PAO4l-H									
CuAO4-C				-0.9					
CuAO4-H									
CuAO4l-C									
CuAO4l-H							0.9		

NOTE: PAO4-like did not show strong +ve or -ve correlation with any form of PAs. Other details are the same as in Supplemental Table 2.

Table S5: Correlation among transcript levels of selected genes with free, conjugated and bound putrescine, spermidine and spermine

Table S4	PUT-F	SPD-F	SPM-F	PUT-C	SPD-C	SPM-C	PUT-B	SPD-B	SPM-B
OAT1-H							0.9		
PEPC-C			-0.8			-0.8			
PEPC-H							0.9		
CDH-C					-0.8		0.8		-0.9
CDH-H		0.9							
Lhcb1-C									
Lhcb1-H	0.9	0.9	-0.8	0.9	0.9	-0.9		0.9	
Lhcb2-C									
Lhcb2-H	0.9		-1.0	1.0	0.9	-0.9		1.0	
Lhcb3-C									
Lhcb3-H									
Lhcb4-C									
Lhcb4-H									
Lhcb5-C									
Lhcb5-H		1.0							-0.8
Lhcb6-C									
Lhcb6-H		1.0							-0.8
psbA-C	0.9	-0.9	-0.9		-0.8	-0.9		0.8	
psbA-H									-1.0
accD-C									
accD-H							1.0		
HSP17.6-H			-0.9	0.9	0.9	-0.9		0.9	
HSP20.0-H	0.8		-1.0	1.0	0.9	-0.9		0.9	
HSP20.1-H	0.9		-0.9	0.8	0.8			0.8	
CBF1-C							-0.8		0.9
CBF2-C				0.8					0.8
CBF3-C				0.9					

NOTE: *Lhcb3* and *Lhcb4* did not show strong +ve or -ve correlation with any Form of PAs. Other details are the same as in Supplemental Table 2.

Table S6: Correlation among transcript levels of PA metabolic genes

Table S5	*ARG1*	*ARG2*	*ADC1*	*ADC2*	*ODC1*	*ODC2*	*AIH1*	*CPA*	*SAMDC1*	*SAMDC2*	*SAMDC3*	*SPDS1*	*SPDS2*	*SPMS*	*PAO2*	*PAO4like*	*CuAO4*	*CuAO4like*
ARG1-C	1.0	0.8												1.0			0.8	
ARG1-H	1.0					0.9	0.9	0.8				0.8		-0.9		0.8	0.8	
ARG2-C	0.8	1.0																
ARG2-H		1.0						0.8						-0.9				
ADC1-C			1.0	1.0	0.9	-0.8	0.9		1.0	1.0	0.9	1.0	1.0		1.0	0.8		0.9
ADC1-H			1.0															
ADC2-C			1.0	1.0	0.8	-0.8	1.0		1.0	1.0	1.0	1.0	1.0		1.0	0.9		0.9
ADC2-H				1.0	0.8				0.9				1.0		0.9			
ODC1-C			0.9	0.8	1.0	-0.8		0.8	0.8	0.9		0.9	0.8		1.0			
ODC1-H				0.8	1.0								0.8		0.8			
ODC2-C			-0.8	-0.8	-0.8	1.0			-0.8	-0.9			-0.8		-0.8	-0.8		
ODC2-H	0.9					1.0												0.8
AIH1-C			0.9	1.0			1.0		1.0	0.9	1.0	0.9	1.0		0.8	0.9		1.0
AIH1-H	0.9						1.0	0.9			0.8	0.9		-0.8		0.8	0.9	
CPA-C					0.8			1.0										
CPA-H	0.8	0.8					0.9	1.0			0.8	1.0		-0.9			1.0	
SAMDC1-C			1.0	1.0	0.8	-0.8	1.0		1.0	1.0	1.0	1.0	1.0		0.9	0.9		0.9
SAMDC1-H				0.9					1.0				0.9		1.0	0.8		
SAMDC2-C			1.0	1.0	0.9	-0.9	0.9		1.0	1.0	0.9	1.0	1.0		1.0	0.8		0.9
SAMDC2-H										1.0								0.9
SAMDC3-C			0.9	1.0			1.0		1.0	0.9	1.0	0.9	1.0		0.8	0.9		1.0
SAMDC3-H							0.8	0.8			1.0	0.9					0.9	
SPDS1-C			1.0	1.0	0.9		0.9		1.0	1.0	0.9	1.0	1.0		0.9			0.9
SPDS1-H	0.8						0.9	1.0			0.9	1.0					1.0	
SPDS2-C			1.0	1.0	0.8	-0.8	1.0		1.0	1.0	1.0	1.0	1.0		0.9	0.9		1.0
SPDS2-H				1.0	0.8				0.9				1.0		0.9			
SPMS-C	1.0													1.0		-0.8	0.9	
SPMS-H	-0.9	-0.9					-0.8	-0.9						1.0				
PAO2-C			1.0	1.0	1.0	-0.8	0.8		0.9	1.0	0.8	0.9	0.9		1.0			0.8
PAO2-H				0.9	0.8				1.0				0.9		1.0			
PAO4l-C			0.8	0.9		-0.8	0.9		0.9	0.8	0.9		0.9	-0.8		1.0	-0.9	0.9
PAO4l-H	0.8						0.8		0.8							1.0		0.8
CuAO4-C	0.8													0.9		-0.9	1.0	
CuAO4-H	0.8						0.9	1.0			0.9	1.0					1.0	
CuAO4l-C			0.9	0.9			1.0		0.9	0.9	1.0	0.9	1.0		0.8	0.9		1.0
CuAO4l-H						0.8				0.9						0.8		1.0

Table S7: Correlation among transcript levels of PA metabolic genes and selected genes

Table S6	*ARG1*	*ARG2*	*ADC1*	*ADC2*	*ODC1*	*ODC2*	*AIH1*	*CPA*	*SAMDC1*	*SAMDC2*	*SAMDC3*	*SPDS1*	*SPDS2*	*SPMS*	*PAO2*	*PAO4like*	*CuAO4*	*CuAO4like*
OAT1-C	0.9	1.0						0.8						0.8				
OAT1-H						0.8				0.9						0.8		1.0
PEPC-C		0.9																
PEPC-H										0.9								1.0
CDH-C	0.8	0.9						1.0										
CDH-H				1.0	0.9				0.9				1.0		0.9			
Lhcb1-C	-1.0						0.8							-1.0		0.8	-0.8	0.8
Lhcb1-H	-0.8													0.8				
Lhcb2-C	-1.0						0.8							-0.9		0.8	-0.8	0.8
Lhcb2-H	-0.9	-0.9					-0.8	-0.8						1.0				
Lhcb3-C	-1.0	-0.9												-1.0				
Lhcb3-H	-0.8													0.8				
Lhcb4-C	-1.0	-0.8												-1.0		0.8	-0.8	
Lhcb4-H				0.8							0.8		0.8					
Lhcb5-C	-1.0	-0.8						-0.8						-1.0			-0.8	
Lhcb5-H				0.8									0.9					
Lhcb6-C	-0.9	-0.9						-0.9						-0.9				
Lhcb6-H				0.8	0.8								0.9					
psbA-C			0.9	0.9	0.8	-1.0	0.9		0.9	0.9	0.8	0.8	0.9		0.9	0.9		0.8
psbA-H											0.8							
accD-C			0.8	0.9		-0.8	0.9		0.9	0.8	0.9		0.9	-0.8		1.0	-0.9	0.9
accD-H						0.9				0.8								1.0
HSP17.6-H	-0.8	-0.9	0.8		0.8			-0.8						0.9				
HSP20.0-H	-0.9	-0.9					-0.8	-0.9						1.0				
HSP20.1-H	-0.8	-0.9					-0.8	-0.8				-0.8		0.9			-0.8	
CBF1-C								-1.0										
CBF2-C								-0.9										
CBF3-C								-0.8										

Table S8: Correlation among transcript levels of genes selected for comparison

Table S7	*OAT1*	*PEPC*	*CDH*	*Lhcb1*	*Lhcb2*	*Lhcb3*	*Lhcb4*	*Lhcb5*	*Lhcb6*	*psbA*	*accD*	*HSP17.6*	*HSP20.0*	*HSP20.1*	*CBF1*	*CBF2*	*CBF3*
OAT1-C	1.0	0.8	0.9	-0.8	-0.8	-0.9	-0.9	-0.9	-1.0						-0.8	-0.8	
OAT1-H	1.0	1.0									0.9						
PEPC-C	0.8	1.0															
PEPC-H	1.0	1.0									0.9						
CDH-C	0.9		1.0			-0.8		-0.8	-1.0						-1.0	-0.9	
CDH-H			1.0				0.8	0.8	0.9								
Lhcb1-C	-0.8			1.0	1.0	0.9	1.0	0.9	0.8		0.8						
Lhcb1-H				1.0	0.9	1.0		0.8	0.8			0.8	0.8				
Lhcb2-C	-0.8			1.0	1.0	0.9	1.0	0.9	0.8		0.9						
Lhcb2-H				0.9	1.0	0.9						0.9	1.0	0.9			
Lhcb3-C	-0.9		-0.8	0.9	0.9	1.0	1.0	1.0	1.0						0.8	0.8	
Lhcb3-H				1.0	0.9	1.0		0.9	0.8			0.8	0.8				
Lhcb4-C	-0.9			1.0	1.0	1.0	1.0	0.9	0.9		0.8						
Lhcb4-H			0.8				1.0	1.0	0.9	0.8							
Lhcb5-C	-0.9		-0.8	0.9	0.9	1.0	0.9	1.0	0.9						0.8	0.9	0.8
Lhcb5-H			0.8	0.8		0.9	1.0	1.0	1.0								
Lhcb6-C	-1.0		-1.0	0.8	0.8	1.0	0.9	0.9	1.0						0.9	0.9	
Lhcb6-H			0.9	0.8		0.8	0.9	1.0	1.0								
psbA-C										1.0	0.9						
psbA-H							0.8			1.0							
accD-C				0.8	0.9		0.8			0.9	1.0						
accD-H	0.9	0.9									1.0						
HSP17.6-H				0.8	0.9	0.8						1.0	1.0				
HSP20.0-H				0.8	1.0	0.8						1.0	1.0	0.8			
HSP20.1-H					0.9								0.8	1.0			
CBF1-C	-0.8		-1.0			0.8		0.8	0.9						1.0	1.0	0.9
CBF2-C	-0.8		-0.9			0.8		0.9	0.9						1.0	1.0	1.0
CBF3-C								0.8							0.9	1.0	1.0

Table S9: Statistically significant differences (p-value) among heat response genes expression studies and polyamines accusation

Category	Time Points→	0h vs ½ h	0h vs 1h	0h vs 6h	0h vs 12h	0h vs 24h
Genes	*SlARG1*	0.04*	0.02*	0.004**	0.01*	0.003**
	SlARG2	0.09ns	0.006**	0.001***	0.02*	0.01*
	SlADC1	<0.0002***	<0.0005***	<0.0004****	<0.0229*	<0.0004****
	SlADC2	<0.0001****	<0.0003***	<0.0003****	<0.0001****	<0.0001***
	SlODC1	<0.0001	0.0001	0.0002	<0.0001	0.0001
	SlODC2	<0.0001****	0.0008***	0.0007***	0.0037**	0.0010***
	SlAIH	<0.0001****	0.0115*	0.0004***	0.0231*	0.0001***
	SlCPA	0.0420*	0.0018**	0.0028**	0.0533ns	0.0061**
	SlSAMDc1	0.0008***	0.0331*	0.1658ns	0.0012**	0.6017ns
	SlSAMDc2	0.0008***	0.0004***	<0.0001****	<0.0001****	0.3354ns
	SlSAMDc3	0.0128*	0.9989ns	0.0015**	0.0569ns	>0.9999ns
	SlSPDS1	<0.0023**	<0.0115*	<0.0053**	<0.0320*	<0.0405*
	SlSPDS2	<0.0004***	<0.0004***	<0.0027**	<0.0030 **	<0.0103*
	SlSPMS	0.9826ns	0.6504ns	0.0022**	0.0010**	0.0053**
	SlPAO2	0.0523ns	0.2542ns	0.3383ns	0.0779ns	0.1060ns
	SlPAO4-like	0.0036**	0.0053**	0.0065**	0.0943ns	0.0012**
	SlCuAO4	0.5500ns	0.0013**	0.0050**	0.1331ns	0.0050**
	SlCuAO4-like	0.0013**	0.0008***	0.0059**	0.1028ns	0.9945ns

Metabolite	Time Points→	0h vs ½ h	0h vs 1h	0h vs 6h	0h vs 12h	0h vs 24h
Free	PUT	0.1369ns	0.0668ns	0.0385*	0.0159*	0.0265*
	SPD	0.0493*	0.0444*	0.1747ns	0.0350*	0.0218*
	SPM	0.0073**	0.3288ns	0.0532ns	0.0118*	0.0088**
Conjugated	PUT	0.0391*	0.6638ns	0.0511ns	0.0539ns	0.0471*
	SPD	0.0142*	0.1403ns	0.0319*	0.0141*	0.0052**
	SPM	0.2263ns	0.1777ns	0.9148ns	0.9907ns	0.9063ns
Bound	PUT	0.8082ns	0.9791ns	0.4184ns	0.0635ns	0.3709ns
	SPD	0.2420ns	0.6366ns	0.0056**	0.0099**	0.0076**
	SPM	0.3671 ns	0.3213 ns	0.0335*	0.7978 ns	0.6318 ns

Table S10: Statistically significant differences (p-value) in cold response time points among genes expression studies and polyamines accusation

Genes	Time Points→	0h vs 1h	0h vs 6h	0h vs 12h	0h vs 24h
PUT Biosynthesis	*SlARG1*	0.0452*	0.0003***	0.04746^{ns}	0.0121*
	SlARG2	0.0796^{ns}	0.0260*	0.0027**	0.0100**
	SlADC1	0.0343*	0.0051**	0.0041**	0.0083**
	SlADC2	0.0048**	0.0007***	0.0007***	<0.0001****
	SlODC1	0.1114^{ns}	0.0823^{ns}	0.0012**	0.0024**
	SlODC2	0.3323^{ns}	0.0189*	0.0018**	<0.0001****
	SlAIH	0.0171*	0.3971^{ns}	0.5436^{ns}	0.0003***
	SlCPA	>0.9999^{ns}	0.0075**	0.0527^{ns}	0.1940^{ns}
SPD & SPM Biosynthesis	*SlSAMDc1*	0.0078**	0.0005***	0.0042**	0.0008***
	SlSAMDc2	0.0046**	0.0017**	0.0061**	0.0024**
	SlSAMDc3	0.0204*	0.0035**	0.0204*	0.0003***
	SlSPDS1	0.8702^{ns}	0.0342*	0.0518 ^{ns}	0.0109*
	SlSPDS2	0.0662^{ns}	0.0038**	<0.0001****	0.0007***
	SlSPMS	<0.0001****	0.0278*	0.0023**	0.0070**
PA catabolism	*SlPAO2*	0.0583^{ns}	0.0005***	0.0016**	0.0012**
	SlPAO4-like	0.0525^{ns}	<0.0001****	0.0058**	0.0014**
	SlCuAO4	0.1206^{ns}	0.5992^{ns}	0.0270*	0.5992^{ns}
	SlCuAO4-like	0.0088**	0.0019**	0.0017**	0.0053**

Specific PA Forms	PA Types	0h vs 1h	0h vs 6h	0h vs 12h	0h vs 24h
Free	PUT	0.6068^{ns}	0.1098^{ns}	0.3443^{ns}	0.0425*
	SPD	0.9005^{ns}	0.7849^{ns}	0.9291^{ns}	0.5837^{ns}
	SPM	>0.9977^{ns}	0.7260^{ns}	0.5981^{ns}	0.3933^{ns}
Conjugated	PUT	0.0511^{ns}	0.0004***	0.1331^{ns}	0.0233*
	SPD	0.9954^{ns}	0.9933^{ns}	0.5858^{ns}	0.2497^{ns}
	SPM	0.9980^{ns}	0.3040^{ns}	0.3271^{ns}	0.4728^{ns}
Bound	PUT	0.2132^{ns}	0.0485**	0.9799^{ns}	0.0027**
	SPD	0.1446^{ns}	0.9999^{ns}	0.8558^{ns}	0.3788^{ns}
	SPM	0.2439^{ns}	0.3255^{ns}	0.1420^{ns}	0.9747^{ns}

Figure S1: qRT-PCR of genes that encode proteins associated with photosynthesis such as nuclear encoded light-harvesting chlorophyll a/b-binding genes *LHCB1-6* and plastid encoded PSII complex gene psbA and accD genes in response to heat stress.

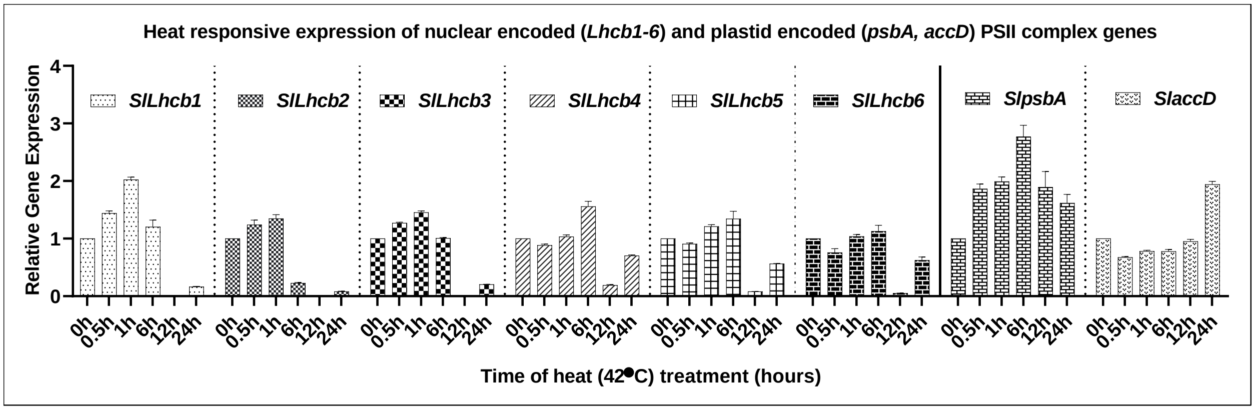

Figure S2: qRT-PCR of genes which encode proteins associated with photosynthesis such as nuclear encoded light-harvesting chlorophyll a/b-binding genes *LHCB1-6* and plastid encoded PSII complex genes psbA and accD genes in response to cold stress.

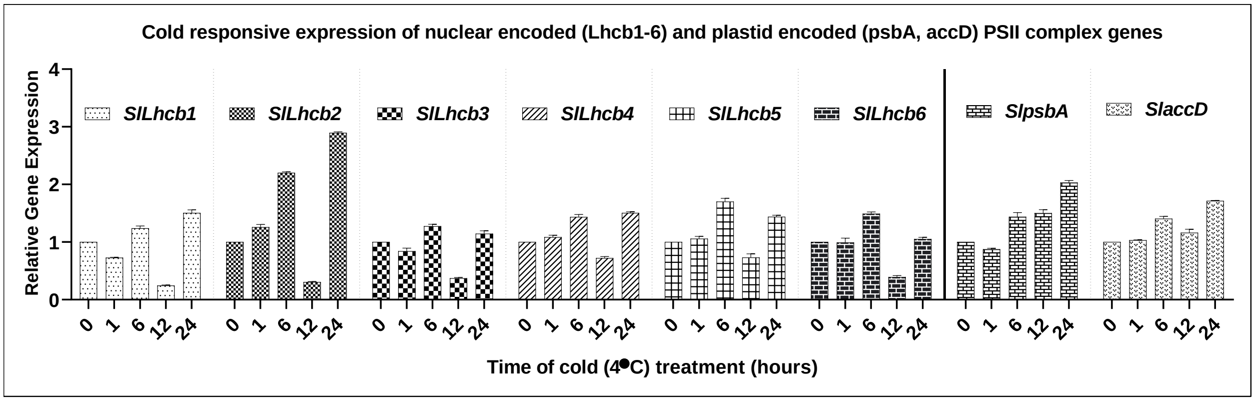

Figure S3: qRT-PCR of gene expression of phosphoenolpyruvate carboxylase (*PEPC*) and Isocitrate dehydrogenase (*ICDH*) genes in response to heat or cold stress

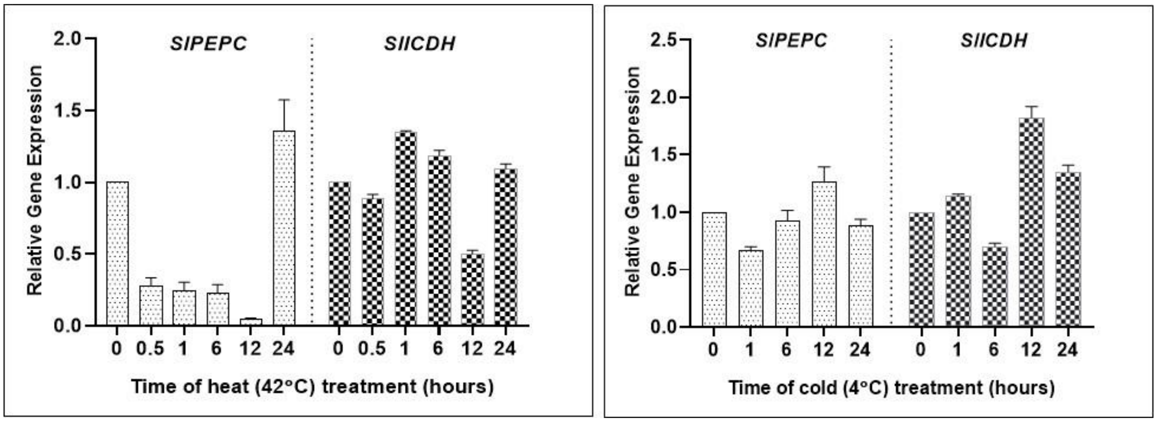

Figure S4: qRT-PCR gene expression of ornithine decarboxylase (*OAT1)* gene in response to heat or cold stress

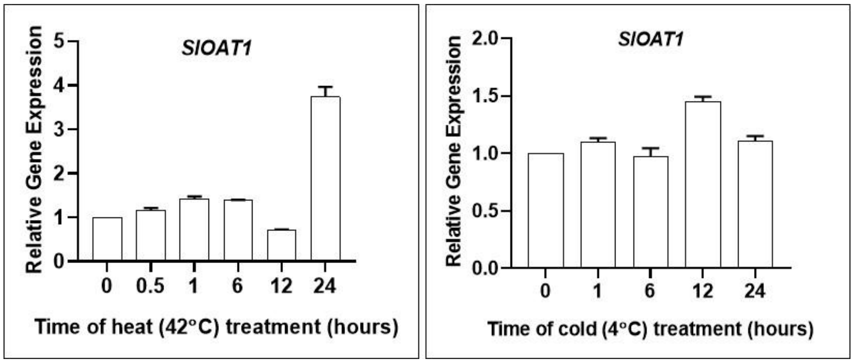

Figure S5: PCA analyses of active and variable parameters during heat stress. The active parameters included heat-stressed seedlings at 0.5, 1, 6, 12 and 24 h stress. The variable parameters included the levels of free (F), conjugated (C) and bound (B) PUT, SPD and SPM (PUT-F, SPD-F, SPM-F, PUT-C, SPD-C, SPM-C, PUT-B, SPD-B and SPM-B). Transcript levels of PAs biosynthesis and catabolizing enzymes genes, heat stress marker genes and photosynthesis-related genes. Before PCA analyses, all values for active and variable parameters were normalized as per cent 0-time values. Other details were the same as stated in the material and method section. Genes abbreviations are as in the TableS1. PCA analyses was performed using XLSTAT (https://www.xlstat.com).

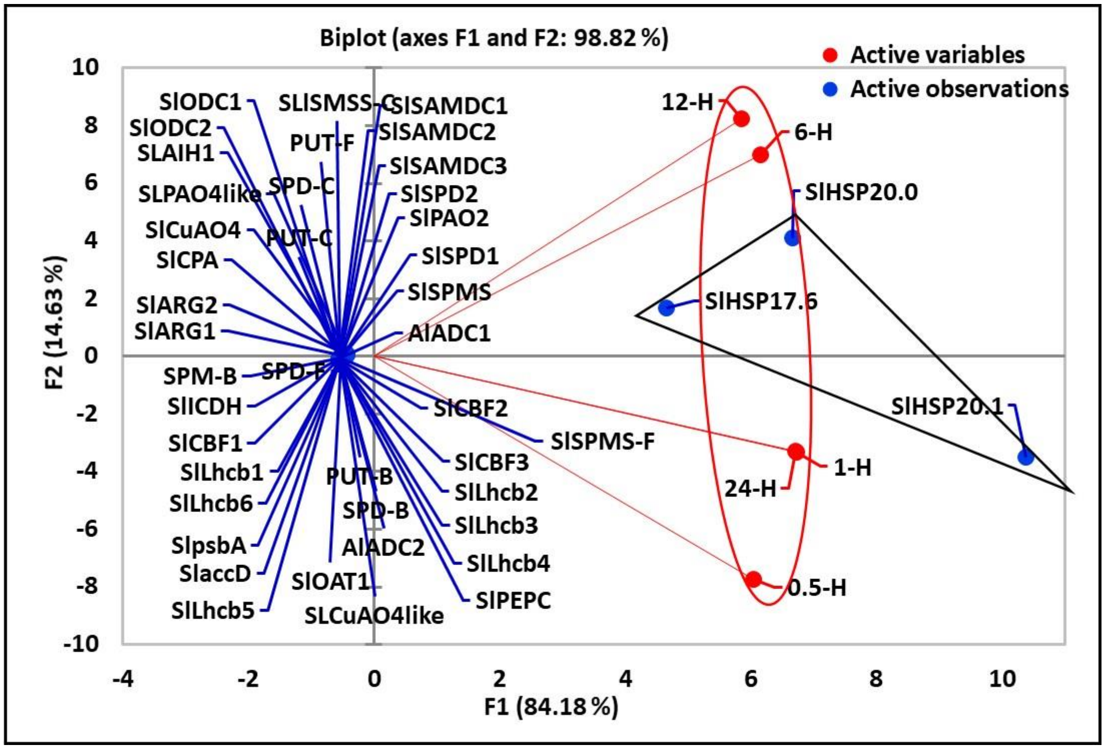

Figure S6: PCA analyses of active and variable parameters during cold stress. The active parameters included cold stressed seedlings at 1, 6, 12 and 24h stress. The variable parameters included the levels of free (F), conjugated (C) and bound (B) PUT, SPD and SPM (PUT-F, SPD-F, SPM-F, PUT-C, SPD-C, SPM-C, PUT-B, SPD-B and SPM-B). Transcript levels of PAs biosynthesis and catabolizing enzymes genes, cold stress marker genes and photosynthesis related genes. Before PCA analyses, all values for active and variable parameters were normalized as per cent 0-time values. Other details as stated in the material and method section. Gene abbreviations are the same as in the TableS1. PCA analyses was performed using XLSTAT (https://www.xlstat.com).

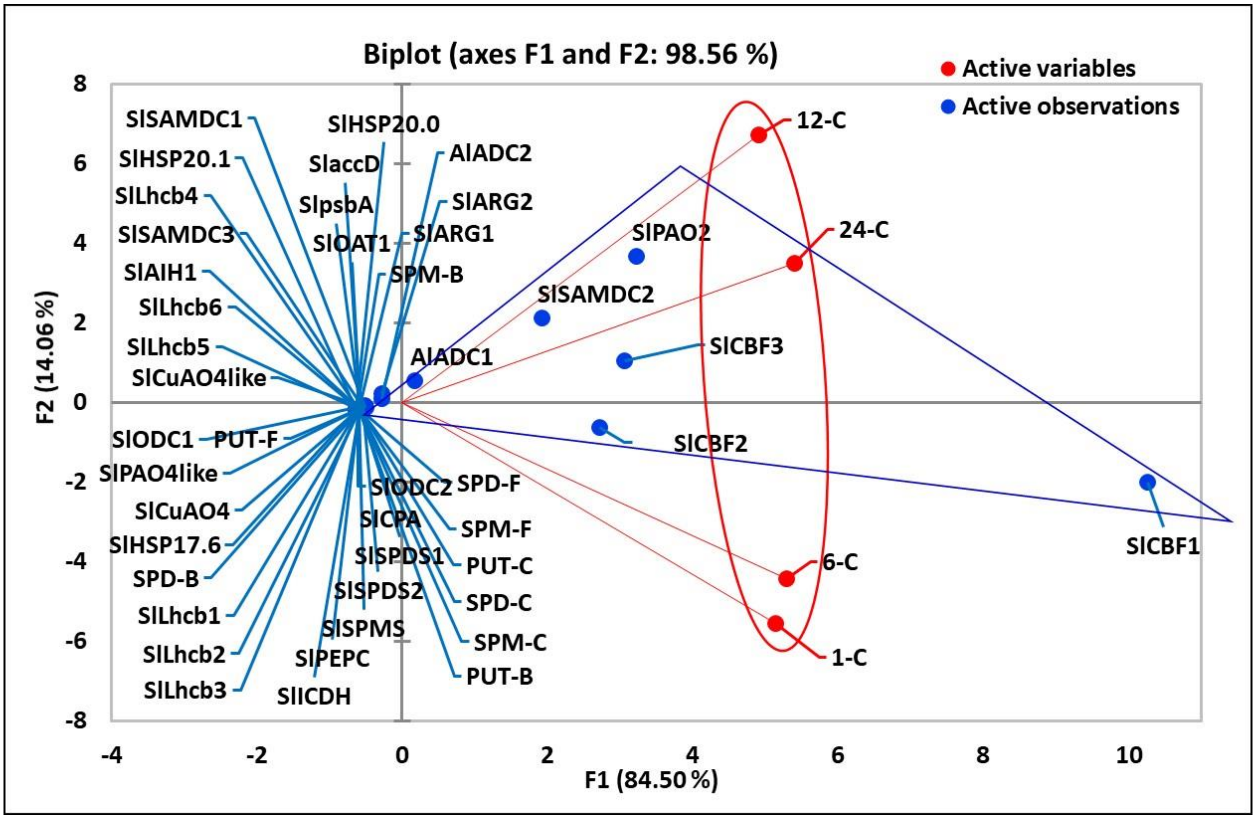

Figure S7: Cytoscape analyses of Pooled data from heat stress

All values for the active and the variable parameters were normalized as % initial 0 time point. Before determining correlation coefficient *r,* the correlation coefficients were determined using Microsoft EXCEL program. These data were analyzed using Expression Correlation APP of Cytoscape program ((Shannon et al., 2003). Gene abbreviations are given in the Supplemental Table 1.

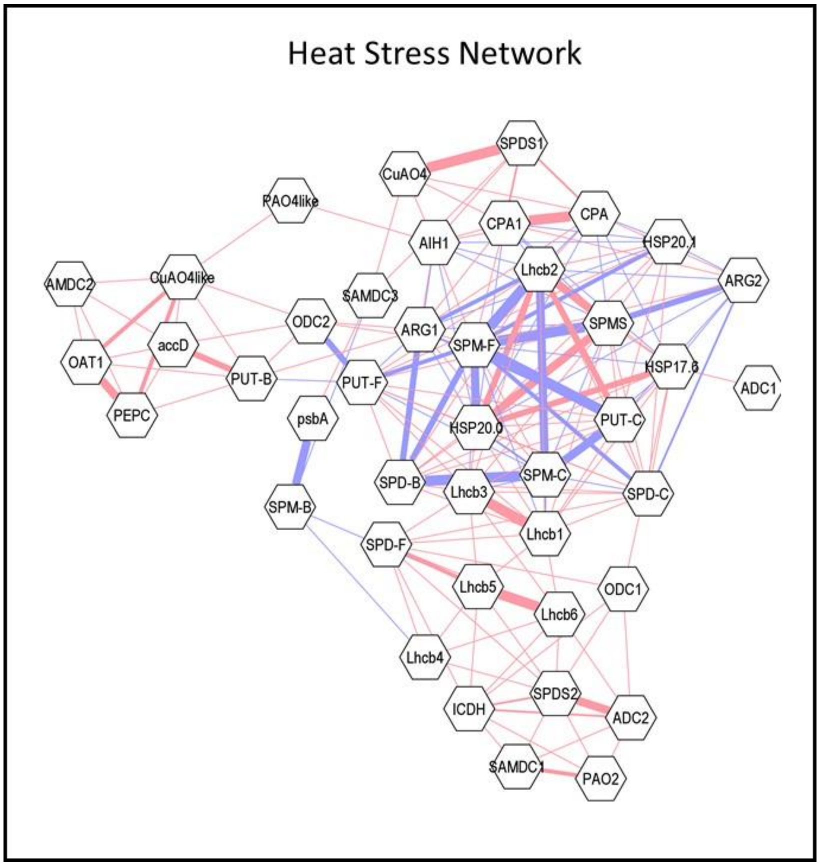

Figure S8: Cytoscape analyses of Pooled data from cold stress

All values for the active and the variable parameters were normalized as % initial 0 time point. Before determining correlation coefficient *r*, the correlation coefficients were determined using Microsoft EXCEL program. These data were analyzed using Expression Correlation app of Cytoscape program (Shannon et al., 2003). Genes abbreviations are the same as in Table S1.

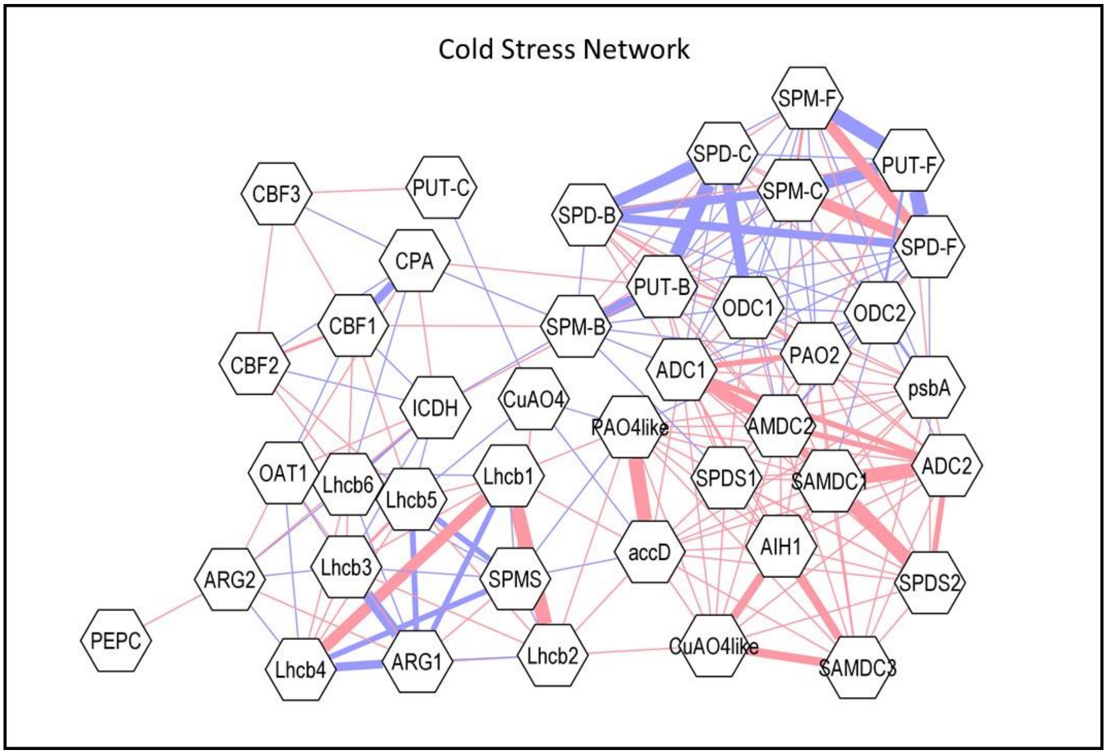

Supplement: Supplementary file 1 [file cells-09-01749-s001.pdf]
